# Supplementary material for: A 7T interleaved fMRS and fMRI study on visual contrast dependency in the human brain
Source: Imaging Neurosci (Camb). 2023 Nov 17;1:imag-1-00031. doi: 10.1162/imag_a_00031 (PMC12007529; doi:10.1162/imag_a_00031)

Supplementary Material

Supplementary Table 1. MRSinMRS checklist. Description of MRS data acquisition, analysis, and quality assessment.

MRSinMRS checklist	
1. Hardware	
a. Field Strength	7T
b. Manufacturer	Philips
c. Model	Achieva
d. RF coil	Quadrature transmit/32-channel receive head coil (Nova Medical)
e. Additional hardware	Dielectric pad (Webb et al. 2022)
2. Acquisition	
a. Pulse sequence	Interleaved sLASER and 3D-EPI
b. Volume of interest	V1
c. Nominal VOI size	14 x 31 x 14 mm
d. Repetition time (TR), echo time (TE)	TR/TE=3600/36 ms
e. Total number of acquisitions/averages	224
i. Number of averaged spectra per time point	n.a.
ii. Averaging method	None, dynamic spectral-temporal fitting
f. Additional sequence parameters	3000 Hz; 1024 data points
g. Water suppression method	VAPOR
h. Shimming method	HOS-DLT (Boer et al. 2020)
i. Triggering or motion correction method	None
3. Data analysis methods and outputs	
a. Analysis software	FSL-MRS v2.1.6
b. Processing steps deviating from quoted product	Processing using in-house Matlab scripts (coil combine, ecc, spectral registration)
c. Output measure	Dynamic fitting on unscaled spectra
d. Quantification references and assumptions	Default basis set
4. Data quality	
a. Reported variables	SNR, FWHM
b. Data exclusion criteria[1]	Metabolites not evaluated when >50% subjects >20% CRLB
c Quality measures of post processing model fitting	Glu CRLB, NAA FWHM
d. Sample spectrum	Figure 1

Supplementary Figure 1. The specified models in FSL-MRS.

Note that the FSL-MRS model constrains all Lorentzian line broadening and shift (eps) terms to be the same across metabolites, unless specifically separated. In this study only macromolecules were freed to have their own shift and broadening terms.

<u>Fixed model</u>

The concentrations are modeled using a GLM, specifically the concentration of each metabolite is constrained to be a linear combination of scaled regressors, where the scaling factors are the beta terms (‘stim0’, ‘stim1’, ‘drift_1’, ‘drift_2’, ‘constant’) and the regressors are the columns of the design matrix. All other parameters (gamma = Lorentzian broadening, sigma = Gaussian broadening, eps = metabolite frequency shifts, baseline = Nth order polynomial baseline, Phi_0 = zero-order phase, Phi_1 = first-order phase) are fixed to take a single value across all time points. Two bounds are imposed: the beta term corresponding to the constant regressor is constrained to positive values or zero, similarly the Lorentzian broadening term (gamma) is also constrained to positive values or zero.

```
# Parameter - functional relationships
Parameters = {
'conc'     : {'dynamic':'model_glm_conc','params':['stim0', 'stim1', 'drift_1', 'drift_2', 'constant']},
'gamma'    : 'fixed',
'sigma'    : 'fixed',
'eps'      : 'fixed',
'baseline' : 'fixed',
'Phi_0'    : 'fixed',
'Phi_1'    : 'fixed'
}

# Bounds on free fitted parameters
Bounds = {
'gamma' : (0,None),
'constant': (0, None)}

# Dynamic models
from numpy import dot

# concentration model
def model_glm_conc(p, t):
    return dot(t[:, [0, 1, 2, 3, 4]], p)

# concentration model gradients
def model_glm_conc_grad(p, t):
    return t[:, [0, 1, 2, 3, 4]].T
```

<u>Variable linewidth model</u>

The concentrations are modeled using a GLM, specifically the concentration of each metabolite is constrained to be a linear combination of scaled regressors, where the scaling factors are the beta terms

('stim0', 'stim1', 'drift_1', 'drift_2', 'constant') and the regressors are the columns of the design matrix. Additionally, the Gaussian line broadening is also modeled as a GLM. The design matrix contains regressors specific to concentrations, with stimulation periods modeled using a constant RECT function, and to sigma (BOLD-induced line broadening), with stimulation periods modeled using the glover HRF. All other parameters (gamma = Lorentzian broadening, eps = metabolite frequency shifts, baseline = Nth order polynomial baseline, Phi_0 = zero-order phase, Phi_1 = first-order phase) are fixed to take a single value across all time points. Two bounds are imposed: the beta term corresponding to the constant regressor is constrained to positive values or zero, similarly the Lorentzian broadening term (gamma) is also constrained to positive values or zero.

```
# Parameter - functional relationships
Parameters = {
'conc'     : {'dynamic':'model_glm_conc','params':['stim0', 'stim1', 'drift_1', 'drift_2', 'constant']},
'gamma'    : 'fixed',
'sigma'    : {'dynamic':'model_glm_sigma','params':['stim0', 'stim1', 'drift_1', 'drift_2', 'constant']},
'eps'      : 'fixed',
'baseline' : 'fixed',
'Phi_0'    : 'fixed',
'Phi_1'    : 'fixed'
}

# Bounds on free fitted parameters
Bounds = {
'gamma' : (0,None),
'constant': (0, None)}

# Dynamic models
from numpy import dot

# concentration model
def model_glm_conc(p, t):
    return dot(t[:, [0, 1, 4, 5, 6]], p)

# concentration model gradients
def model_glm_conc_grad(p, t):
    return t[:, [0, 1, 4, 5, 6]].T

# sigma models
def model_glm_sigma(p, t):
    return dot(t[:, [2, 3, 4, 5, 6]], p)

# sigma model gradients
def model_glm_sigma_grad(p, t):
    return t[:, [2, 3, 4, 5, 6]].T
```

Supplementary Table 2. Statistical results for the fixed linewidth full-block analysis.

	10%		100%		mean activation		100 vs 10%	
	z	p	z	p	z	p	z	p
Metabolite level								
Asc	0.83	0.20	0.28	0.39	0.67	0.25	-0.34	0.37
Asp	-1.51	0.07	-1.86	0.03	**-2.02**	**0.02**	-0.30	0.38
GSH	0.38	0.35	1.51	0.07	1.25	0.11	0.88	0.19
Glu	**2.58**	**0.005**	**3.46**	**<0.001**	**3.45**	**<0.001**	1.19	0.11
Ins	-0.09	0.46	**2.35**	**0.01**	1.27	0.10	-1.86	0.03
PE	-1.12	0.13	0.30	0.38	-1.01	0.16	1.07	0.14
Scyllo	0.45	0.33	-0.01	0.50	0.29	0.38	-0.34	0.37
Glc+	**-2.16**	**0.01**	**-2.57**	**0.005**	**-3.02**	**0.001**	-0.38	0.35
tCh	1.24	0.11	-0.49	0.31	0.51	0.30	-1.19	0.12
tCr	0.52	0.30	**2.67**	**0.003**	**2.55**	**0.005**	1.59	0.05
tNAA	**-1.89**	**0.03**	0.86	0.20	-0.66	0.26	1.61	0.05

Positive and negative z-values indicate a neurometabolite increase and decrease, respectively, in case of the 10%, 100% and mean activation. For the contrast between 10% vs 100% a positive z-value indicates 100% > 10% , whereas a negative z-value indicates 10% > 100%. The significant effects (p<0.05) are displayed in bold.

Supplementary Table 3. Statistical results for the fixed linewidth sub-block analysis.

	10%		100%		mean activation		100 vs 10%	
	z	p	z	p	z	p	z	p
Metabolite level								
Asc	0.61	0.27	-0.08	0.47	0.37	0.36	-0.52	0.30
Asp	-1.26	0.10	-0.19	0.42	-1.07	0.14	0.81	0.21
GSH	0.44	0.33	0.85	0.20	0.87	0.19	0.30	0.38
Glu	**2.08**	**0.02**	**3.48**	**<0.001**	**2.92**	**0.002**	0.54	0.30
Ins	-0.71	0.24	**2.05**	**0.02**	0.71	0.24	1.94	0.03
PE	**-2.16**	**0.02**	0.85	0.20	-1.33	0.09	1.75	0.04
Scyllo	-0.20	0.42	-0.62	0.27	-0.56	0.29	-0.31	0.38
Glc+	-0.91	0.18	**-1.99**	**0.02**	**-1.97**	**0.02**	-0.85	0.20
tCh	**2.06**	**0.02**	-0.30	0.38	1.27	0.10	-1.71	0.04
tCr	-0.15	0.44	**2.09**	**0.02**	1.47	1.07	1.49	0.07
tNAA	**-2.03**	**0.02**	0.86	0.20	-0.83	0.20	1.70	0.04

Positive and negative z-values indicate a neurometabolite increase and decrease, respectively, in case of the 10%, 100% and mean activation. For the contrast between 10% vs 100% a positive z-value indicates 100% > 10% , whereas a negative z-value indicates 10% > 100%. The significant effects (p<0.05) are displayed in bold.

Supplementary Table 4. Correlations between fMRI and fMRS

	10%		100%		100 vs 10%	
	r	*p*	*r*	*p*	*r*	*p*
Full-block						
fMRI BOLD vs fMRS Glu	0.46	0.04	-0.03	0.91	0.24	0.31
fMRI BOLD vs fMRS sigma	-0.29	0.22	0.11	0.64	-0.005	0.98
Sub-block						
fMRI BOLD vs fMRS Glu	0.33	0.16	-0.02	0.94	0.34	0.14
fMRI BOLD vs fMRS sigma	-0.39	0.09	0.19	0.43	-0.07	0.76

Supplementary Figure 2. Correlations between fMRI and fMRS

Scatterplots of the fMRI BOLD signal (x-axis) and the glutamate (top rows) or the sigma, i.e. the linewidth changes (bottom rows) for the 10%, 100% and 100>10% conditions. A linear regression line through the individual data points is shown with the 95% confidence intervals in dotted lines.

Full-block

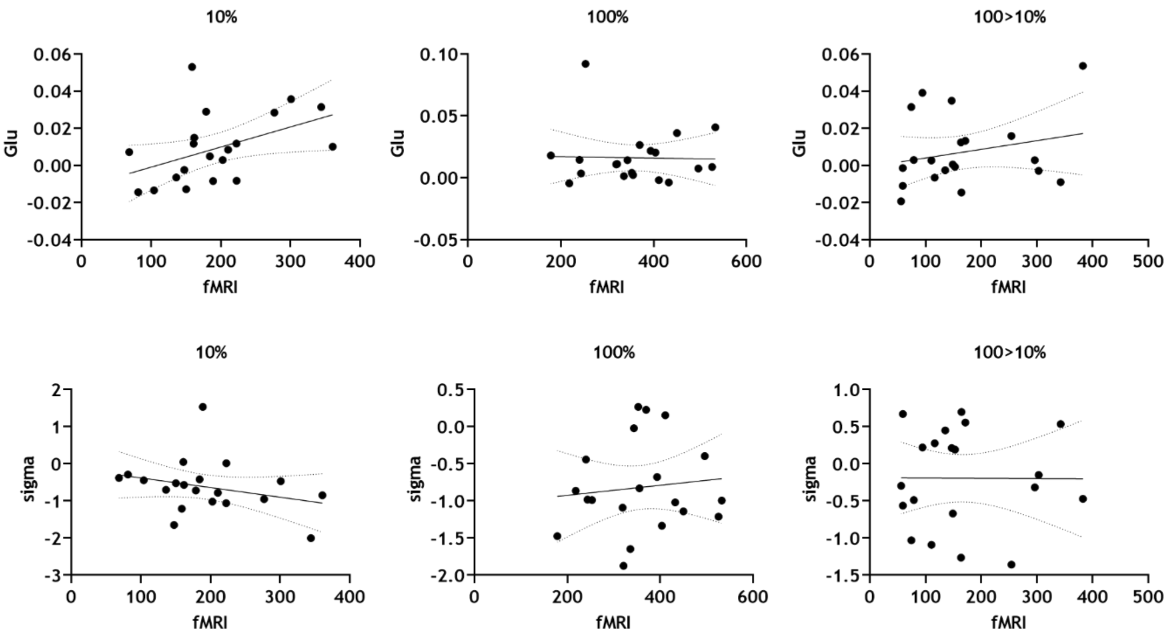

Sub-block

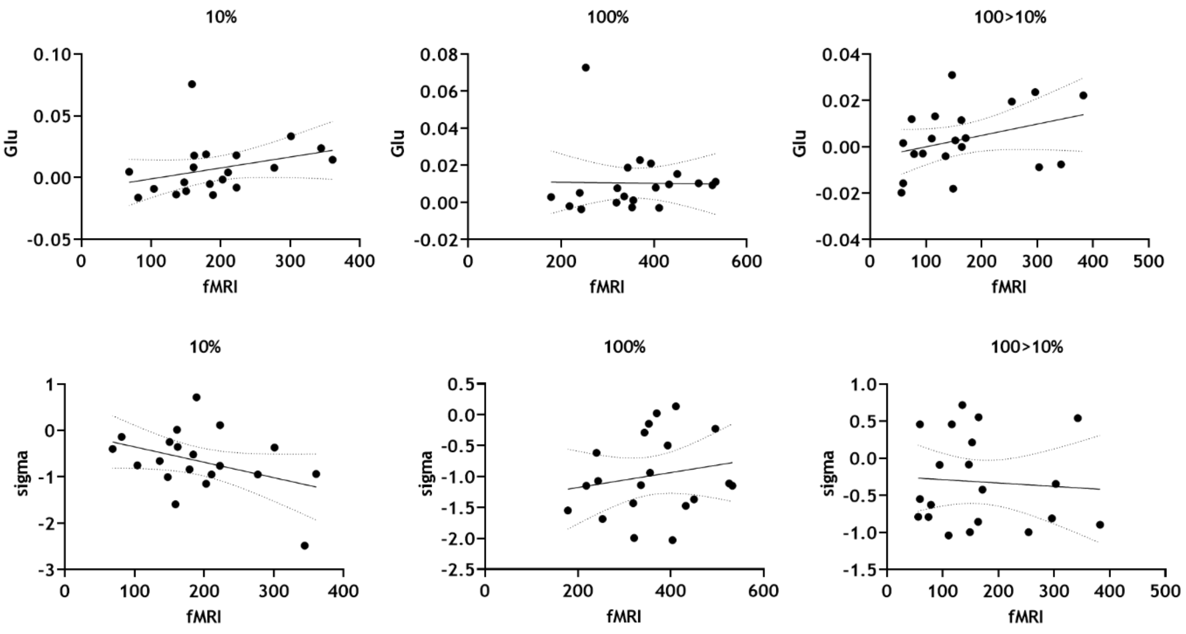

Supplement: Supplementary Material [file imag_a_00031-supp.pdf]
